# Extraction and Characterization of *N*,*N*‑Dimethyltryptamine from *Mimosa tenuiflora*: A Multivariate Approach

**DOI:** 10.1021/acsomega.5c06560

**Published:** 2025-09-16

**Authors:** Lucas Cordeiro de Oliveira, Taynah Pereira Galdino, Marcelo da Silva Pedro, Mateus Araujo Luz, Igor de Melo Castro, Evilasio Anisio Costa Filho, João Davi da Silva Gonçalves, Antonio Gilson B. Lima, Victor Ignacio Afonso, Marcus Vinicius Lia Fook, Suédina Maria de Lima Silva

**Affiliations:** † Northeast Biomaterials Evaluation and Development Laboratory, CERTBIO, Academic Unit of Materials Engineering, 154624 Federal University of Campina Grande, Campina Grande 58429-900, Brazil; ‡ Academic Unit of Medicine, Federal University of Campina Grande, Campina Grande 58400-398, Paraíba, Brazil; § Department of Mechanical Engineering, Federal University of Campina Grande, Campina Grande 58429-900, Brazil; ∥ Academic Unit of Physics, Federal University of Campina Grande, Campina Grande 58429-900, Paraíba, Brazil

## Abstract

*N*,*N*-Dimethyltryptamine (DMT), a plant-derived tryptamine alkaloid, has attracted growing interest due to its therapeutic potential in treating mental health disorders resistant to conventional pharmacological interventions. This study aimed to establish an efficient methodology for the extraction, isolation, and characterization of DMT, and to identify the most viable portion of the *Mimosa tenuiflora* plant (root bark vs stem bark) through a multianalytical approach to assess the biomedical applicability of the isolated compound. Samples were subjected to various characterization techniques and methodological analyses. Among the tested samples, sample 2C—obtained using methodology 2, which employed the stem bark—yielded 3.45% (calculated from 5.0003 g of powdered stem bark, corresponding to approximately 0.172 g of pure DMT) and exhibited a robust phytochemical profile, with a significant presence of alkaloids, tannins, and flavonoids. Morphological characterization by scanning electron microscopy (SEM) revealed a heterogeneous, amorphous surface, whereas recrystallization produced well-defined prismatic crystals. Elemental composition, evaluated by energy-dispersive X-ray spectroscopy (EDS) and X-ray fluorescence (XRF), revealed a high proportion of carbon (76.03%) and nitrogen (23.97%), along with trace elements typical of plant matrices, such as calcium and iron. Fourier-transform infrared spectroscopy (FTIR) showed characteristic absorption bands of indole functional groups, confirming the presence of DMT. Thermogravimetric analysis (TGA) demonstrated thermal stability up to approximately 135 °C—a critical parameter for pharmaceutical processing. DMT identification was confirmed by high-performance liquid chromatography with diode-array detection (HPLC-DAD), showing a retention time of 11.81 min and absorbance peaks at 275, 280, and 288 nm, consistent with this alkaloid. Gas chromatography–mass spectrometry (GC–MS) further validated the identity, yielding a retention time of 16.4 min and 88% spectral similarity with the NIST library, including characteristic fragments at *m*/*z* 58, 130, and 188. The cellular viability of the isolated DMT exceeded 85% at therapeutic concentrations, with a significant reduction observed only at 100 μg/mL (53 ± 21%), possibly due to experimental overexposure. These findings identify sample 2C as a promising candidate for the development of standardized pharmaceutical formulations containing DMT and provide robust analytical support for future standardization, scale-up, and clinical application within the framework of psychedelic-assisted psychotherapy.

## Graphical abstract

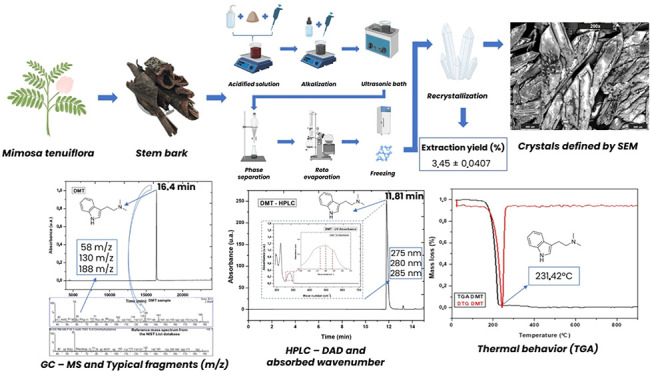

## 1 Introduction

*N*,*N*-Dimethyltryptamine (DMT), a substituted tryptamine, is an alkaloid consisting of an indole core linked to an ethylamine side chain bearing a dimethylated nitrogen. Its chemical structure is relatively stable due to the indole moiety, whereas the nitrogen-containing side chain is susceptible to chemical transformations such as oxidation, deamination, and methylation. The indole ring facilitates interactions with biological receptors, particularly serotonergic targets, while the *N*,N-dimethyl substitution critically enhances the binding affinity of DMT to these receptors, underlying its potent psychoactive effects.^1^

The molecular structure of the DTM, present in various plant species such as *Banisteriopsis caapi*, *Psychotria viridis*, *Anadenanthera peregrina*, *Anadenanthera colubrina*, and *Mimosa tenuiflora*, is responsible for eliciting psychedelic effects when it is administered at appropriate concentrations.^2–4^ This compound exhibits high structural similarity to serotonin and is capable of interacting with the same serotonergic receptors, particularly those of the 5-HT receptor family. This interaction is largely attributed to the distinctive chemical features of DMT compared with common tryptamines—namely, the presence of two methyl groups attached to the nitrogen atom of the amine moiety. This modification enhances receptor affinity and increases its lipophilicity, both of which are pharmacological properties that contribute to the compound’s efficacy within the central nervous system.^5,6^ These properties—particularly lipophilicity—enable DMT to cross the blood–brain barrier and act as a serotonergic agonist, predominantly at 5-HT2A receptor in key brain regions such as the hippocampus, amygdala, and neocortex.^7^ At sufficient concentrations in the brain, DMT can induce alterations in perception, visual distortions, shifts in consciousness, emotional processing, and cognition. Furthermore, it appears to enhance functional connectivity between brain regions that typically exhibit limited communication, thereby increasing neural networks entropy. These effects position DMT as a promising candidate for the treatment of mental health disorders such as anxiety and depression.^8^

When administered orally, DMT′s bioavailability is reduced, as the compound is readily degraded by monoamine oxidase (MAO) enzymes present in the gastrointestinal tract (GIT).^9^ Therefore, for effective activity, it is common to coadminister DMT with MAO inhibitors (MAOIs), as practiced by indigenous Amazonian peoples who combined *Psychotria viridis*, rich in DMT, with *Banisteriopsis caapi*, which contains harmine, harmaline, and tetrahydroharmine—β-carbolines capable of inhibiting MAO.^10^

Besides its presence in the plants commonly used for ayahuasca brew preparation, DMT occurs at concentrations of approximately 2% in the stem bark and root bark of *Mimosa tenuiflora* (Wild.) Poiret, an endemic plant of the Caatinga biome in northeastern Brazil. Popularly known as “Jurema preta”, this plant is considered sacred in Afro-Indigenous syncretic religions and is used in various rituals in the form of infusions or Jurema “wines”. However, these preparations do not produce psychedelic effects in consumers because they are not coadministered with MAOIs. Consequently, nearly all DMT is inactivated in the gastrointestinal tract, since these extractions are typically performed using only water and heat, which do not efficiently isolate DMT.^11–13^

The use of native plant species to extract pharmacologically relevant biomolecules, such as DMT, in response to a rapidly increasing global demand, raises concerns about ecological impacts, particularly when plant sacrifice is involved. However, this does not necessarily lead to resource depletion, as many of these species are fast-growing, adapted to local conditions, and can be sustainably propagated through reforestation and cultivation.^14^ In particular, *M. tenuiflora* is highly adaptable to semiarid environments due to its deep root system and abundant seed production throughout the year, which enables survival and proliferation in nutrient-poor soils with low water availability.^15,16^ Indeed, *M. tenuiflora* is a pioneer species that colonizes degraded areas and plays a significant role in soil restoration.^17^ It also serves as an indicator of progressive secondary succession or ecological recovery, often being the only woody species present.^18^

The efficient extraction of DMT from *M. tenuiflora* has been the subject of significant methodological advancements. As is well documented from ancient indigenous use, studied by anthropologists—see for instance Grünewald^19^—and from modern scientific literature, DMT is present at much lower concentrations in the leaves and flowers (0.01–0.03% dry weight) compared to the barks, which contain approximately 2 orders of magnitude more, while the seeds show no significant alkaloid content.^20,21^ Some studies^15^ indicate that the root bark is the preferred matrix, with DMT content ranging from 0.5% to 1.7%, surpassing the concentrations typically found in stem bark, which are approximately 0.3%. Therefore, our work will focus on the barks. It is also important to note that, in obtaining the relevant plant parts (stem and root barks), there is no need to sacrifice the entire individual, as only portions of the bark and roots are required and can be adequately removed for use in the methodological process.

Among the most commonly employed methods for extracting DMT from this matrix is acid–base extraction, which involves the initial use of acidic solvents (pH ∼ 2), followed by basification and the application of nonpolar solvents such as *n*-hexane, diethyl ether, and dichloromethane, as described by Gaujac et al.^22^ Alternative methodologies include the use of strongly basic solutions (pH ∼ 14) with sodium hydroxide combined with organic solvents such as ethyl acetate and *n*-butanol.^23^ Another approach involves neutral-medium extraction over a 10 h period, followed by solid-phase extraction (SPE) using buffer and methanol.^4^ Operational variables, including temperature (40–60 °C), extraction time, agitation speed (rpm), and the application of ultrasound, can also significantly influence process efficiency.

However, no standardized methodology exists for extracting DMT from *M. tenuiflora*, which affects process reproducibility and highlights opportunities for research aimed at optimizing the parameters described in the literature. Therefore, this study aimed to evaluate different conditions and methodologies for DMT extraction from *M. tenuiflora*, a species chosen for its widespread availability in northeastern Brazil, as well as to assess the efficiency of the process in isolating the DMT compound and its potential for biomedical applications.

## 2 Experimental Section

### 2.1 Materials, Reagents, and Equipment

The stem and root bark of *M. tenuiflora* were manually collected in the municipality of Juazeirinho (Paraíba state, Brazil). The samples were identified and stored in a dry place at room temperature (25 °C) until processing. Analytical-grade (P.A.) reagents used included sodium hydroxide (Neon), hydrochloric acid (ACS Scientific), sodium carbonate (Dynamics), *n*-hexane (ACS Scientific), and calcium chloride (Nuclear). Ultrapure water was obtained using a Milli-Q system (Merck). Consumables such as qualitative filter paper, voile fabric, and laboratory glassware (beakers, Erlenmeyer flasks and separatory funnels) were used as required.

The equipment employed comprised a drying oven (Solab), knife mill (IKA), 150-mesh sieves (100 μm), magnetic stirrer (IKA), ultrasonic bath (Unique), rotary evaporator (Fisatom), pH meter (OHAUS), and analytical balance (OHAUS).

### 2.2 Raw Material Preparation

The stem and root bark were washed with distilled water and dried in an oven at 50 °C for 24 h. After drying, the material was ground in a knife mill at 10,000 rpm until a fine powder was obtained. The powder was subsequently sieved, and fractions smaller than 150 mesh were selected, in accordance with the specifications of the Brazilian Pharmacopoeia for semifine particles.

### 2.3 Evaluation of Extraction Methodologies

Three distinct methodologies were evaluated for the extraction and purification of target compounds, followed by physicochemical characterization (pH, density, moisture content, ash content, and sulfated ash content), phytochemical analysis, X-ray fluorescence (XRF), and determination of extraction yield. The objective was to identify the most suitable method for performing thermal (TGA) and spectroscopic (FTIR) analyses, as well as confirmatory techniques including high-performance liquid chromatography with diode array detection (HPLC-DAD), gas chromatography–mass spectrometry (GC–MS), and cellular viability assessment via cytotoxicity assays.

#### 2.3.1 Methodology 1

Adapted from Pantrigo,^24^ this method involved dissolving 4 g of powdered material in 0.5 M NaOH (0.1 g/mL) under magnetic stirring at 25 °C for 24 h, protected from light. After filtration through voile fabric, the filtrate underwent liquid–liquid partitioning with *n*-hexane (2.5:1 ratio), followed by ultrasonication for 30 min and separation in a separatory funnel; this procedure was repeated twice. The organic layer was dried with calcium chloride (∼30 g), vacuum filtered, evaporated in an oven at 50 °C to a final volume of 20 mL, and cooled to −10 °C for crystallization.

#### 2.3.2 Methodology 2

In Methodology 2, adapted from Moreira^25^ and illustrated in the flowchart in Figure 1, 5 g of powdered material was dissolved in 0.2 M HCl (50 mL) under stirring at 700 rpm and 60 °C for 1 h, followed by vacuum filtration. The filtrate underwent three defatting steps with *n*-hexane (20 mL each, 30 min ultrasonication), and the hexane phase was discarded. The resulting solution was then basified to pH 12 with NaOH and stirred at 700 rpm for 24 h at room temperature, protected from light. The product was extracted with *n*-hexane (20 mL, three repetitions, 30 min ultrasonication), and the combined hexane fractions were concentrated using a rotary evaporator and cooled to −10 °C for crystallization.

**Figure 1:**
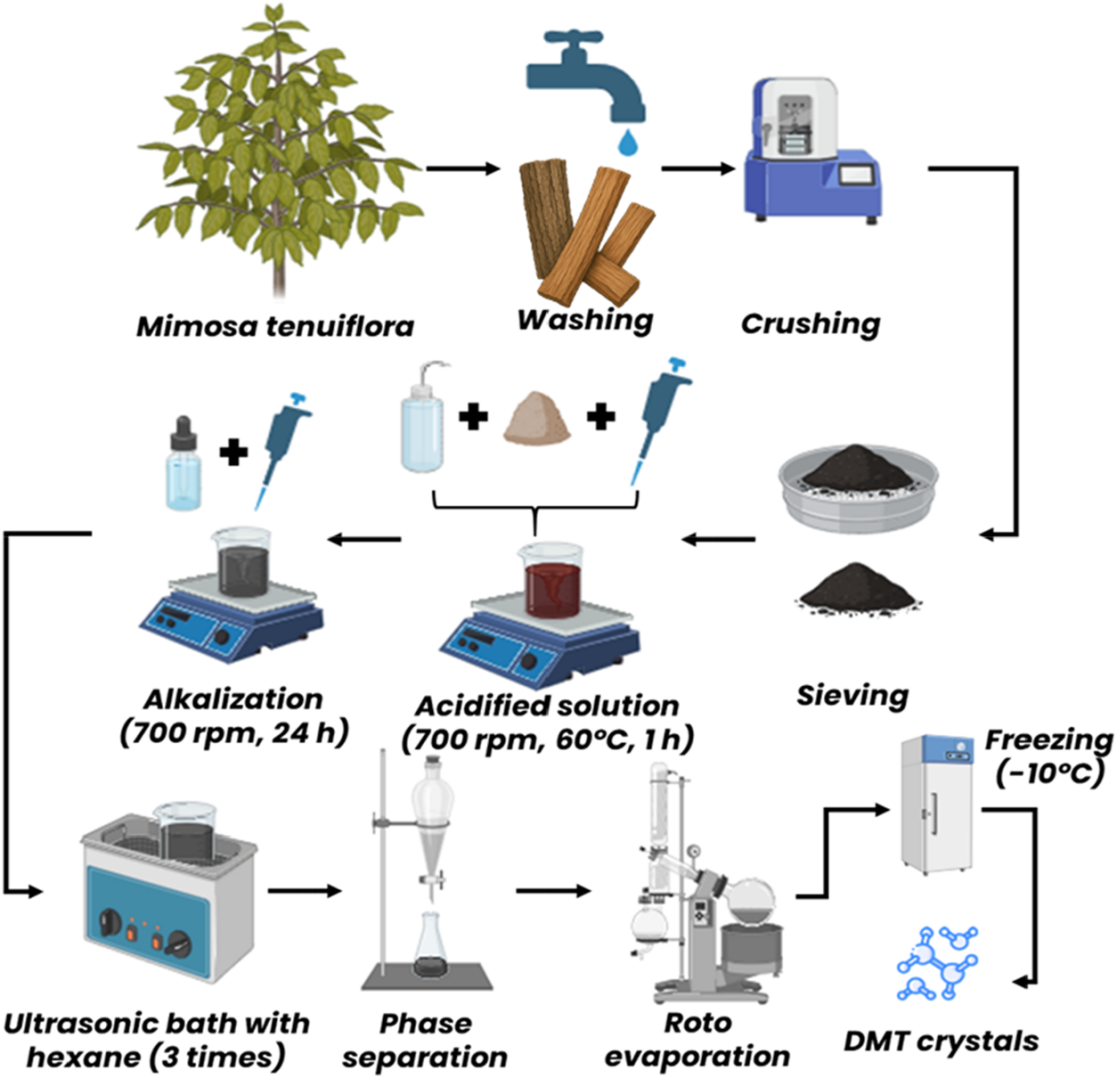
Flowchart illustrating the methodology applied in the DMT extraction and crystallization process. Original image by the author. Copyright 2025, Oliveira, L. C.

After pulverization, the plant material was acidified with 0.2 M HCl to protonate the amines present in the alkaloids and increase their solubility in the aqueous medium, allowing the removal of lipophilic impurities via extraction with *n*-hexane. The solution was then alkalinized with NaOH to pH 12, converting the amines into their free base forms and facilitating selective extraction with *n*-hexane. The parameters of temperature, time, and stirring were based on Moreira,^25^ with adjustments made from preliminary tests to optimize extraction efficiency and preserve DMT integrity.

#### 2.3.3 Methodology 3

Adapted from Gaujac,^26^ this procedure involved extracting 5 g of powdered material in 0.1 M HCl (100 mL) under stirring for 24 h at room temperature, protected from light, followed by vacuum filtration. The filtrate was defatted with *n*-hexane (2 × 20 mL, 30 min ultrasonication). The pH was then adjusted to 11 with NaOH and further increased to 12 with Na_2_CO_3_, and the final extraction was performed with *n*-hexane (2 × 20 mL, 30 min ultrasonication). The combined hexane phases were evaporated and cooled to −10 °C for crystallization.

#### 2.3.4 Sample Identification on the Basis of Methodology and Matrix

The extracts were named and organized as shown in Table 1. Among the tested methodologies, Method 2 exhibited the highest yield and purity, with advantages including shorter extraction time, improved pH control, and greater efficiency in removing interfering substances. Consequently, Method 2 was selected for subsequent characterization.

**Table 1:** Nomenclature Used to Identify the Extractions Performed

sample	methodology	plant matrix
1C	methodology 1	stem bark
1R	methodology 1	root bark
2C	methodology 2	stem bark
2R	methodology 2	root bark
3C	methodology 3	stem bark
3R	methodology 3	root bark

### 2.4 Performed Analyses

#### 2.4.1 Physicochemical Analyses

The pH was measured using a properly calibrated bench pH meter, with the sample dispersed in distilled water. Density was determined using the pycnometer method, which accounts for the sample mass and displaced volume. Moisture content was assessed by drying the sample in an oven at 100 °C until a constant weight was achieved. Ash content was determined by calcining the sample in a muffle furnace at 600 °C, allowing quantification of the residual inorganic material. Sulfated ash content was determined by adding concentrated sulfuric acid to the previously calcined sample, followed by a second calcination at 800 °C.

#### 2.4.2 Phytochemical Analysis

Qualitative tests for alkaloids, flavonoids, saponins, tannins, terpenes, and other secondary metabolites were performed on aqueous and alcoholic extracts obtained from the samples, following the protocols described by ref 27. Precipitation reactions or color changes were interpreted as positive results.

#### 2.4.3 X-ray Fluorescence (XRF)

Powdered samples (<150 mesh) were pressed into pellets, and analyzed using a Malvern Panalytical Epsilon 4 energy-dispersive X-ray fluorescence (XRF) spectrometer. Elemental analysis covered the range from carbon (C) to ammonium (NH_4_^+^), with detection limits from subppm levels up to 100 wt %. Measurements were performed under a helium atmosphere with an acquisition time of 300 s, operating voltage between 15 and 50 kV, and a current of 1 mA. Quantification was achieved through the internal calibration of the instrument.

#### 2.4.4 Extraction Yield

The extraction yield was determined as the ratio between the mass of DMT crystals obtained and the mass of powdered raw material used. Thus, Yield (%)—the percentage of DMT extracted relative to the initial material—was calculated by dividing the mass of DMT obtained by the mass of raw material used and multiplying the result by 100. This parameter was used to assess process efficiency and to identify the most effective methodology for DMT extraction.

#### 2.4.5 Scanning Electron Microscopy (SEM) and Energy-Dispersive Spectroscopy (EDS)

The samples were mounted on aluminum stubs using carbon conductive tape, and morphological analyses were carried out with a TESCAN VEGA 3 scanning electron microscope (SEM). Imaging was performed at a maximum magnification of 100,000× using backscattered electrons (BSEs) and an acceleration voltage of 15 kV. The SEM was coupled with an energy-dispersive spectroscopy (EDS) system for semiquantitative elemental identification, and spectra were acquired from selected areas through area scans at 2000× magnification.

#### 2.4.6 Fourier Transform Infrared Spectroscopy (FTIR)

Fourier transform infrared analysis was performed using a PerkinElmer Spectrum 400 mid-IR FTIR spectrometer. Samples were placed directly on the diamond crystal and pressed to ensure optimal contact. Spectra were recorded in the range of 4000–400 cm^–1^, with 16 scans at a resolution of 4 cm^–1^. Data processing and spectral analysis were carried out using Origin 8 software.

#### 2.4.7 Thermogravimetric Analysis (TGA)

Thermogravimetric analysis (TGA) was carried out using a TA Instruments Q50 analyzer. Approximately 2 mg of each sample was placed in alumina crucibles and heated from 25 to 800 °C at a rate of 10 °C/min under a nitrogen flow of 50 mL/min. The resulting mass loss curves were recorded and analyzed to assess the thermal stability and decomposition behavior.

#### 2.4.8 High-Performance Liquid Chromatography with Diode Array Detection (HPLC-DAD)

Analyses were performed via an HPLC system coupled with a diode array detector (DAD) employing a PerkinElmer C18 column (5 μm, 150 mm × 4.6 mm). The mobile phase consisted of water with 0.3% formic acid (phase A) and acetonitrile with 0.3% formic acid (phase B), operated under the gradient program: 0–2.4 min (95:5 A/B), 9.6 min (70:30 A/B), and 10.6 min (2:98 A/B); returning to 95:5 A/B at 17.7 min. The volumetric flow rate was 0.5 mL/min, the column was maintained at 30 °C, and the sample compartment at 25 °C. The injection volume was 15 μL, with a total runtime of 18 min. Detection was monitored at 266 nm.

#### 2.4.9 Gas Chromatography–Mass Spectrometry (GC–MS)

Gas chromatography–mass spectrometry analyses were performed via a PerkinElmer Clarus 590 gas chromatograph coupled to a Clarus SQ 8S mass spectrometer, equipped with an Rxi-5Sil MS capillary column (30 m × 0.25 mm × 0.25 μm). Helium was employed as the carrier gas at a flow rate of 1.53 mL/min. Injections were carried out in split mode (1:30), with an injection volume of 1 μL, injector temperature of 250 °C, and a transfer line temperature of 250 °C. The oven temperature program consisted of a ramp of 3 °C/min up to 200 °C (held 2 min), followed by 10 °C/min up to 280 °C (held 2 min). The ion source temperature was set at 200 °C, and electron impact ionization (70 eV) was applied in scan mode (*m*/*z* 40–550). Compound identification was achieved by comparison of mass spectra with the NIST library.

#### 2.4.10 MTT Cytotoxicity Assay

The assay was conducted in accordance with ISO 10993–5:2009 (Biological Evaluation of Medical Devices–Part 5: Tests for In Vitro Cytotoxicity). The L929 fibroblast cell line (ATCC NCTC clone 929) was obtained from the Rio de Janeiro Cell Bank (Brazil). The colorimetric cytotoxicity test used was the 3-(4,5-dimethylthiazol-2-yl)-2,5-diphenyl tetrazolium bromide (MTT) assay, which quantifies mitochondrial dehydrogenase activity as an indicator of metabolic activity, to assess cell viability. Optical density was measured at 570 nm, with a reference wavelength of 650 nm, using a Victor X3 microplate reader (PerkinElmer). Cell viability was expressed as a percentage relative to the control, with outlier detection performed via a modified *z*-score method. A reaction blank was included, and DMT crystals were tested at concentration of 5 μg/mL, 25 μg/mL, 50 μg/mL, 75 μg/mL, 100 μg/mL, and the ISO-recommended 1.2 mg/mL.

## 3 Results and Discussion

### 3.1 Physicochemical Analysis: pH, Density, Moisture Content, Ash Content, and Sulfated Ash Content

Stem and root samples of *M. tenuiflora* were subjected to physicochemical analyses, as summarized in Table 2. The pH values of stem and root bark samples were 5.14 and 4.76, respectively—closely matching the value reported by Lopes et al.^28^ (pH 4.76)—indicate acidic matrices. This acidity is consistent with the presence of secondary metabolites, such as phenolic compounds and tannins, which are common in this type of matrix and can influence the stability and solubility of alkaloids in aqueous media.^29^ Acidic conditions favor alkaloid extraction by promoting DMT protonation and stabilization in its salt form, thereby increasing solubility.

**Table 2:** Physicochemical Analysis of Stem Bark and Root Bark Samples of *M. tenuiflora*

physicalchemical analysis		
analysis	stem bark	root bark
pH	5.14 ± 0.08	4.76 ± 0.10
density (g/cm^3^)	1.0 ± 0.02	1.2 ± 0.04
moisture content (%)	10.32 ± 0.23	8.88 ± 0.01
ash content	1.17 ± 0.27	2.07 ± 0.06
sulfated ash content	2.04 ± 0.17	2.78 ± 0.11

However, a difference in acidity between these two matrices was observed, with the root bark exhibiting slightly higher acidity, possibly due to the presence of secondary metabolites, as also reported by Shi et al.^30^ This increased acidity may affect both the extraction efficiency and the purity of the crystals obtained, since phenolic compounds and tannins can be present at relatively high concentrations.^31^ These metabolites may interact with DMT, hindering its extraction through the formation of complexes with alkaloids via mechanisms such as hydrogen bonding or adsorption. Such interactions can reduce the yield of free alkaloids and complicate their separation from the plant matrix, making the stem bark a more favorable matrix due to its lower acidity, which positively influences the efficiency of the extraction process.

Table 2 also presents the density data for the stem bark (1.0 g/cm^3^) and root bark (1.2 g/cm^3^) of *M. tenuiflora*. Although the difference is relatively small, it may reflect subtle structural variations between the matrices, such as differences in compaction and the relative proportions of structural components, such as cellulose and lignin. While density alone does not fully reveal these characteristics, they can influence bark processing and affect the efficiency of DMT extraction methods. As noted by Jensen, Jørgensen, and Rasmussen,^29^ the structural rigidity of plant matrices can hinder access to bioactive compounds, requiring more effective techniques—such as ultrasonic bath treatment—to promote the disruption of cell walls. In this context, the slightly greater density of the root bark may be a complementary, although not definitive, indication of a more rigid and compact structure, supporting the need for more intensive extraction procedures.

The physicochemical analysis also provided data on moisture content, ash content, and sulfated ash content, with values falling within the acceptable limits for plant-based materials, as reported in the literature. Moisture content was 10.32% for stem bark and 8.88% for root bark, both considerably lower than the approximately 46% reported in *M. tenuiflora* bark samples by Azevêdo et al.^32,33^ Elevated moisture levels can reduce DMT extraction efficiency by increasing the potential for hydrolytic degradation of alkaloids, which are sensitive to water, thereby hindering DMT recovery.

Analysis of ash content indicated a low mineral presence, suggesting minimal contamination. The root bark, however, exhibited slightly higher values (2.07% total ash and 2.78% sulfated ash) compared to the stem bark (1.17% and 2.04% for total and sulfated ash, respectively), consistent with the 1.5–1.8% range reported by Amariz et al.^15^ This difference may be attributed to the root bark’s direct contact with the soil. The discrepancy between the total and sulfated ash contents suggests the presence of sulfated metabolites or external contaminants, which may require additional pretreatment—such as acid washing—to avoid interference in analytical procedures or pharmaceutical applications.

### 3.2 Phytochemical Analysis

Phytochemical analysis of the stem and root barks confirmed the presence of all analyzed groups of secondary metabolites, including saponins, tannins, and phenolic compounds, as summarized in Table 3.

**Table 3:** Phytochemical Profile of *M. tenuiflora* Stem Bark and Root Samples

phytochemical analysis		
analysis	stem bark	root bark
saponins	positive	positive
tannins	positive	positive
phenolic compounds	positive	positive
flavonoids	positive	positive
steroids	positive	positive
triterpenes	positive	positive
alkaloids	positive	positive
quinones	positive	positive
coumarins	positive	positive

The presence of saponins in these plant matrices, which is associated with their foaming properties, is closely linked to spheroidal and triterpenoid structures that confer immunomodulatory, emulsifying, and hemolytic activities. These characteristics can hinder the extraction process, as stable emulsions may form during liquid–liquid partitioning, interfering with efficient phase separation.^34–36^ Additionally, tannins and phenolic compounds were confirmed in the *M. tenuiflora* samples, consistent with expectations for this species. These secondary metabolites are strongly associated with pharmacological activities, including astringent, antioxidant, and anti-inflammatory effects, making them relevant in the medical-pharmaceutical field due to their therapeutic potential. However, tannins can negatively affect alkaloid extraction, as they may form insoluble complexes with alkaloids through hydrogen bonding and hydrophobic interactions.^30,37–40^

These analyses highlight the potential of *M. tenuiflora*, given the presence of additional secondary metabolites confirmed by phytochemical tests. Flavonoids, for example, exhibit anti-inflammatory and antioxidant properties, but tend to create barriers to alkaloids by binding to them via noncovalent interactions, thereby reducing the alkaloid bioavailability.^41,42^ The presence of triterpenes and steroids, possess anti-inflammatory and antitumor properties,^43^ was also confirmed as expected for this species, since they provide structural rigidity to the plant properties.^44,45^ Two other, less abundant groups of secondary metabolites were identified: quinones, which have potential antimicrobial activity,^46^ and coumarins, which may be toxic at high concentrations.^47^

Alkaloids remain the primary focus for therapeutic applications, with their presence confirmed in both stem and root barks, particularly DMT. This finding corroborates the well-documented capacity of *M. tenuiflora* to biosynthesize DMT, as well as potentially other indolic or β-carbolinic alkaloids, as demonstrated in previous chromatographic analyses.^22^ Studies such as those reported by Gaujac et al.^22^ indicate a higher alkaloid concentration in the roots, linking root tissue to elevated DMT levels; however, this also introduces challenges related to purity due to the coextraction of complexing compounds, such as tannins, and potential mineral contaminants present in the roots.

The phytochemical profile of the stem and root barks of *M. tenuiflora* confirms the abundance and diversity of secondary metabolites, reinforcing its pharmacological potential and highlighting the need for specific extraction techniques to selectively isolate alkaloids, as observed by Szmechtyk and Malecka.^48^ This consideration accounts for potential interactions with tannins and other polar metabolites, which may compromise the efficiency of conventional extraction and purification methods.

### 3.3 X-ray Fluorescence (XRF)

The X-ray fluorescence (XRF) analysis data for the stem and root barks are presented in Table 4. Elements such as calcium (Ca), potassium (K), silicon (Si), sulfur (S), and iron (Fe) were detected in varying proportions in both samples, reflecting the influence of soil contact and the geographic region of plant growth, as well as the potential impact these elements on efficiency of alkaloid extraction.

**Table 4:** Elemental Composition (%) of Stem and Root Bark Samples of *M. tenuiflora* Determined by X-ray Fluorescence (XRF)

X-ray fluorescence		
element	stem bark	root bark
Mg	1.52%	1.44%
Al	0.00%	3.78%
Si	2.77%	7.87%
P	2.83%	3.13%
S	2.56%	5.97%
Cl	9.19%	9.36%
K	16.96%	14.66%
Ca	57.26%	30.02%
Ti	0.00%	2.52%
Mn	0.96%	0.36%
Fe	2.45%	17.97%
Zn	0.40%	0.28%
Sr	5.76%	0.50%
Sn	1.19%	0.11%

In both stem and root bark samples, calcium was the element present in the highest proportion, with a greater content in the root bark (57.26%) compared with the stem bark (30.02%). This suggests the possible presence of carbonates from various sources, such as calcium–phenolic complexes, which can influence pH by increasing matrix acidity. The formation of calcium salts of organic acids may reduce the availability of free ionizable groups, potentially interfering with alkaloid extraction by necessitating higher acid quantities for efficient alkaloid protonation. These findings corroborate the previously discussed physicochemical results and are consistent with observations reported by Rizwan et al.^49^

Another element that significantly differed between stem and root barks was iron (Fe), detected at 2.45% and 17.97%, respectively. High concentration iron can accelerate oxidation reactions during the extraction process, consequently reducing the amount yield of DMT. To mitigate the effects of iron on alkaloid extraction, the use of antioxidants or controlled atmospheres may be necessary.

The high calcium content, along with other elements listed in the Table 4, such as iron, may be attributed to the Juazeirinho region (Paraíba, Brazil), where the *M. tenuiflora* samples were collected. This area is known for its mineral-rich soil and is an important mining site for kaolin and iron oxide in Paraíba state, as reported by Queiroz and Morais.^50^

The higher silicon content in the root bark (7.87% compared with 2.77% in the stem bark) suggests the presence of silicon-rich structures that confer greater rigidity to this plant region, corroborating the previously discussed density results. This may require increased energy input and pose methodological challenges during sample processing, emphasizing the importance of more efficient extraction techniques to obtain DMT, such as microwave-assisted extraction. Similarly, sulfur (S), although present in lower amounts, was found in higher percentages in the root bark (5.97% vs 2.56%), likely due to greater soil contact. Sulfur may occur as nutrients, natural compounds, or contaminants, potentially acting as impurities that interfere with the pharmaceutical use of DMT. This requires additional purification steps, thereby increasing the complexity of the extraction process.

Thus, considering the objectives of this investigation in selecting the most viable methodology and plant matrix, the stem bark demonstrates greater potential for the extraction process due to its lower content of catalytic metals and reduced accumulation of minerals that could interfere with the matrix characteristics. These factors, combined with more favorable physicochemical properties such as lower density and ash content, make the stem bark more suitable. Although previous studies have reported relatively high DMT levels in the root bark of *M. tenuiflora*, this study employed a multifactorial evaluation that extends beyond DMT content alone, aiming to identify the optimal plant portion—stem bark or root bark—for DMT extraction. The results indicate that stem bark is the more viable option, a finding that, while less commonly reported in the literature, corroborates the work of Amariz et al.,^51^ who developed a factorial design for DMT extraction from stem bark.

### 3.4 Extraction Yield Analysis

Table 5 presents the data corresponding to the extractions performed using the different combinations of methodology and plant matrix described in Table 1. The table reports the mass of the raw material (g), the mass of DMT crystals (mg) obtained from each extraction, and the corresponding extraction yield (%), allowing comparison of the efficiency of the various approaches.

**Table 5:** Extraction Yield Data for DMT, Emphasizing the Mass of Raw Material Used, the Amount of Crystalline Product Obtained, and the Overall Efficiency of the Extraction Process

sample	raw material (g) (X̅ ± σ)	crystal mass (mg) (X̅ ± σ)	yield (%) (X̅ ± σ)
1C	5.0062 ± 0.0021	146.08 ± 4.93	2.91 ± 0.0977
1R	5.0014 ± 0.0006	91.65 ± 1.33	1.83 ± 0.0265
2C	5.0003 ± 0.0001	172.82 ± 2.03	3.45 ± 0.0407
2R	5.0004 ± 0.0001	153.47 ± 2.47	3.07 ± 0.0494
3C	5.0132 ± 0.0061	110.72 ± 1.32	2.20 ± 0.0230
3R	5.0034 ± 0.0051	147.32 ± 1.24	2.94 ± 0.0270

Method 2 demonstrated superior performance, achieving crystal yields above 3%, with the stem bark sample (2C) reaching a yield of 3.45%. This improved performance is likely due to the combined effects of favorable physicochemical conditions, the lower content of calcium and iron compared with the root bark, the multiple purification steps—including the removal of lipids and residual moisture via *n*-hexane—and efficient cell disruption assisted by ultrasonic bath treatment. Additional factors contributing to the enhanced DMT separation of Method 2 included the initial protonation step in an acidic medium using 0.2 M HCl, which promoted the saline solubilization of DMT given the more acidic matrix (pH 4.76–5.14), and the subsequent alkalinization with 0.5 M NaOH, which facilitated the conversion of the protonated amine into a free, lipophilic form.

In contrast, the lower yields observed in Methodologies 1 and 3 can be attributed to less effective acid–base adjustment, reduced efficiency of cell disruption, and limitations in the purification steps. Furthermore, considering the physicochemical data and the higher proportion of potential contaminant elements present in the root bark, along with the lower yield of sample 2R compared with sample 2C in Methodology 2, the stem bark matrix (2C) proved more favorable and was therefore selected for subsequent characterization in this study.

### 3.5 Scanning Electron Microscopy (SEM) and Energy-Dispersive Spectroscopy (EDS)

Figures 2 and 3 present scanning electron microscopy (SEM) images of DMT crystals before and after recrystallization, respectively. Recrystallization was performed by heating the solvent in which the crystals were dissolved, using a fresh portion of *n*-hexane to promote the formation of higher-purity crystals. The samples were characterized using SEM for morphological analysis and energy-dispersive spectroscopy (EDS) for elemental composition.

**Figure 2:**
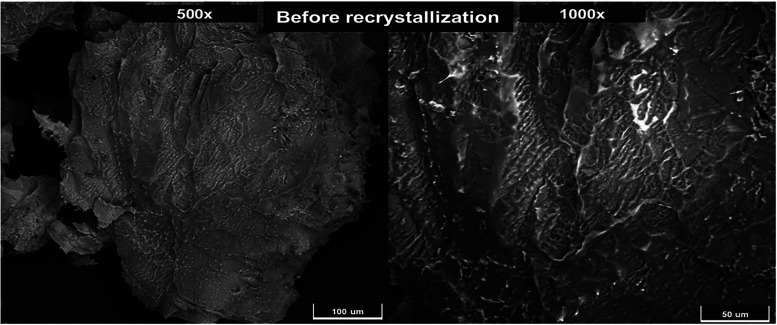
SEM images of DMT crystals prior to recrystallization at 500× (left) and 1000× (right) magnifications.

**Figure 3:**
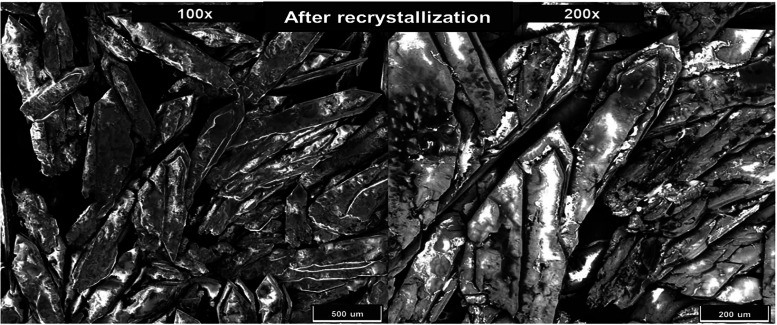
SEM images of DMT crystals after recrystallization at 100× (left side) and 200× (right side) magnifications.

In Figure 2, at 500× and 1000× magnifications, the DMT crystals exhibit an irregular surface morphology, lacking a well-defined crystalline structure. Rough, partially formed surfaces are evident, suggesting incomplete crystal formation. These regions present a disorganized lamellar texture and variable sizes, with crystal contours clearly outlined but edges irregular. This heterogeneous appearance indicates that the material remains in a raw, structurally disordered state. Such morphology is consistent with findings reported by Amariz et al.,^51^ who described DMT crystals with irregular sizes and lamellar structures obtained from the stem bark of *M. tenuiflora*.

The micrographs obtained after recrystallization, shown in Figure 3 at 100× and 200× magnifications, reveal a remarkable transformation in the morphology of the material. The crystals display an elongated, well-defined prismatic structure with sharp edges and a preferential orientation. The dimensions of the recrystallized crystals are considerably larger—exceeding 500 μm in length—and exhibit a more homogeneous and organized arrangement. This morphological reorganization reflects successful purification of the compound, indicating the removal of potential impurities and the formation of a thermodynamically stable crystalline structure. The elongated, translucent appearance of the crystals is characteristic of compounds purified through recrystallization, confirming the effectiveness of the procedure in obtaining DMT with enhanced purity and crystalline order.

In addition to the morphological analysis of the DMT crystals, their elemental composition was evaluated using energy–dispersive X-ray spectroscopy (EDS) coupled with SEM. The quantitative data are presented in Table 6, and the corresponding spectra are shown in Figure 4.

**Figure 4:**
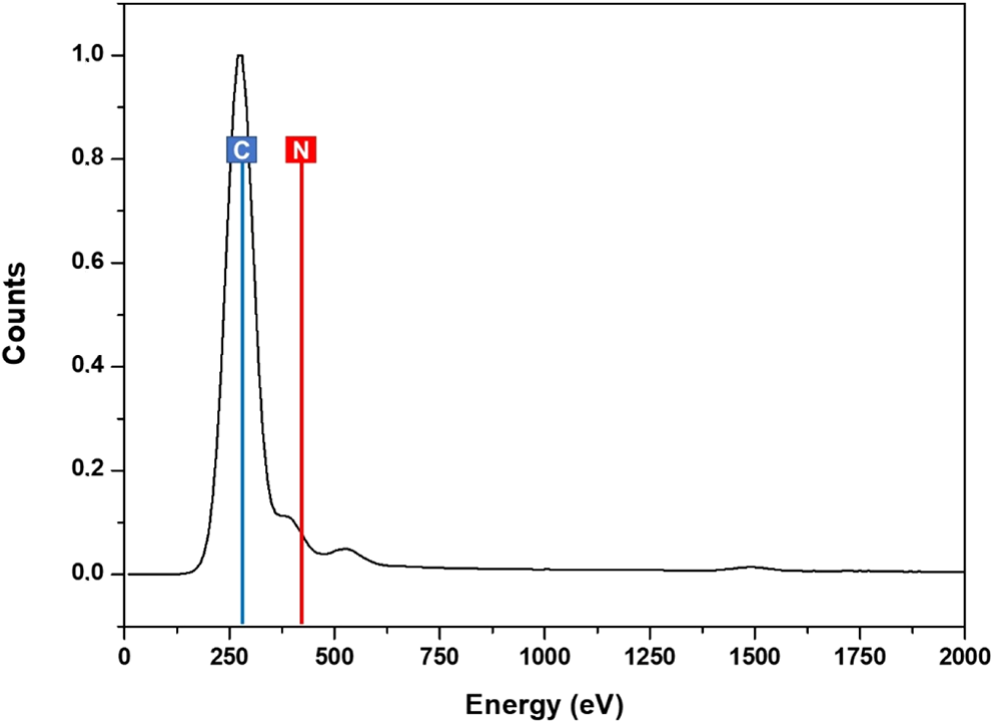
Energy-dispersive X-ray spectroscopy (EDS) of the isolated DMT sample.

**Table 6:** Elemental Composition of the Crystals Determined by EDS Analysis Coupled with SEM

element	%
carbon	76.03
nitrogen	23.97

Figure 4 shows the prominent peaks corresponding to carbon (C) at ∼277 eV and nitrogen (N) at ∼401 eV. The observed high C/N ratio aligns with the expected elemental composition of indole alkaloids, confirming the molecular structure of DMT. The absence of notable peaks from other elements indicates a highly pure isolated compound, with negligible inorganic contamination.

Elemental analysis of the DMT crystals via EDS revealed a composition of 76.03% carbon and 23.97% nitrogen, consistent with the molecular formula C_12_H_16_N_2_, which contains 12 carbon atoms, 2 nitrogen atoms, and 16 hydrogen atoms. Due to the limitation of EDS, which cannot detect elements with atomic number below 4, the presence of hydrogen in the DMT structure cannot be directly confirmed by this method. Nevertheless, the experimentally observed C/N ratio closely matches the theoretical stoichiometry, confirming the identity of the compound and indicating that the crystals consist predominantly of pure DMT, with minimal or no detectable impurities.

The high carbon content is associated with the predominance of aromatic structures and methylated aliphatic chains present in the DMT molecule, while the substantial nitrogen content corresponds to the two amine groups, distinguishing tryptamine-based alkaloids from other classes. The elemental purity confirmed by EDS underscores the effectiveness of the selective extraction process, yielding high-purity DMT. These findings are consistent with previous EDS analysis of alkaloids present in plant bark, such as those reported by Cesari et al.,^52^ and further support the suitability of the purified crystals for pharmacological and bioactivity studies, where precise molecular identity and structural integrity are crucial.

### 3.6 Fourier Transform Infrared Spectroscopy Analysis (FTIR)

Figure 5 presents the FTIR spectrum of the analyzed DMT crystals. The sample exhibits absorption bands at 738.27 cm^–1^, 811.98 cm^–1^, 854.98 cm^–1^, 1001.28 cm^–1^, 1105.99 cm^–1^, and 1173.00 cm^–1^, which are in excellent agreement with literature values (742 cm^–1^, 809 cm^–1^, 862 cm^–1^, 1008 cm^–1^, 1110 cm^–1^, and 1178 cm^–1^, respectively), reported by Gaujac et al.^53^ Minor shifts in frequencies can be attributed to instrumental variations or the crystal’s physicochemical environment, and do not compromise band identification. The bands at 738.27 cm^–1^, 811.98 cm^–1^, and 854.98 cm^–1^ correspond to out-of-plane C–H bonds deformations in substituted aromatic rings, confirming the presence of the indole core, a defining structural feature of DMT.

**Figure 5:**
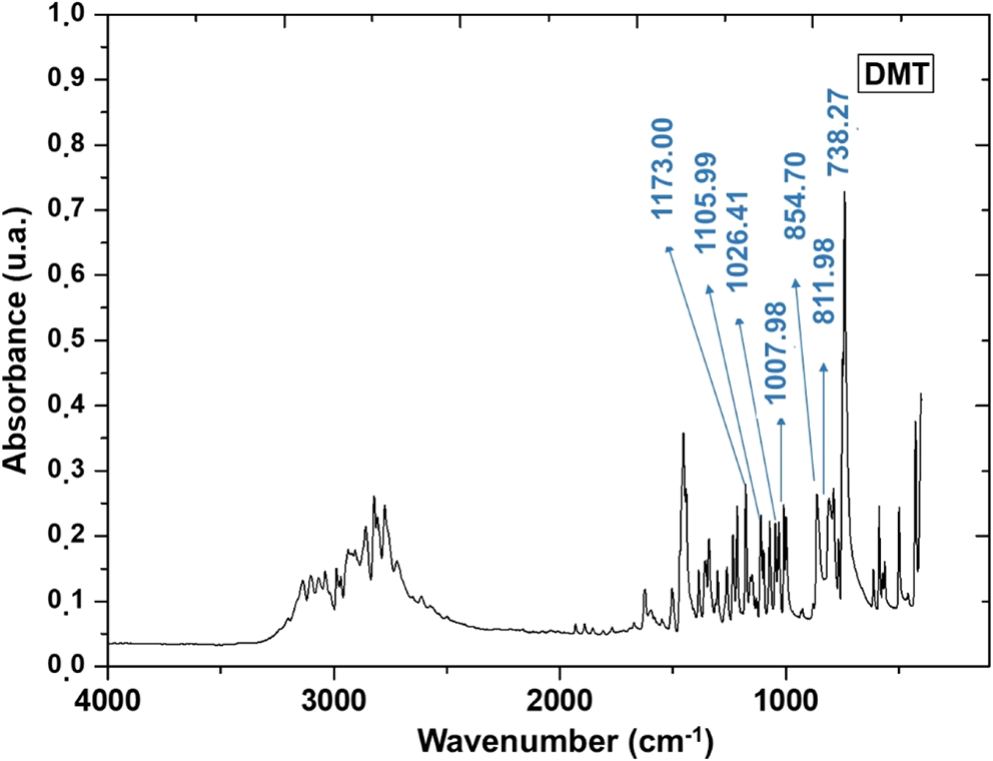
Fourier-transform infrared (FTIR) absorption spectrum of DMT crystals.

The absorption band at 1001.28 cm^–1^ corresponds to in-plane C–H bending vibrations, also attributed to the aromatic system, further corroborating the integrity of the indole ring. Moreover, the bands located at 1105.99 cm^–1^ and 1173.00 cm^–1^ correspond to C–N stretching modes, consistent with the dimethylamine groups present on the molecule’s side chain. These signals are highly diagnostic of the substituted amine functional group and are critical for distinguishing DMT from other structurally related alkaloids. Additionally, the FTIR spectrum displays characteristic N–H bond stretching vibrations around 3400 cm^–1^, confirming the presence of secondary amines, and C–H stretching in the 2850–3000 cm^–1^ range, consistent with aliphatic hydrogens of the dimethylamine moiety. Aromatic C–H stretching near 3050 cm^–1^ and C=C stretching between 1450–1600 cm^–1^ further support the integrity of the indole aromatic framework. The concurrent presence of these N–H, C–H, aromatic C–H, and C=C signals, together with the fingerprint region bands, confirms that the compound retains the complete aromatic and amine functional group profiles reported in the literature. This agreement not only validates the molecular identity of DMT but also indicates high purity and minimal structural alteration during extraction.

The presence and precise positioning of these bands, also observed by Amariz et al.,^51^ strongly indicate the high purity of the DMT crystals obtained via the selected extraction methodology and stem bark matrix (sample 2C), highlighting the efficiency of this extraction process. FTIR spectroscopy, employed here for the accurate compound identification based on characteristic functional groups, is a well-established technique in phytochemistry. For instance, Mishra et al.^54^ effectively used FTIR to characterize the phytoconstituents of *Asparagus racemosus*, successfully identifying multiple compound classes from their unique spectral signatures. Moreover, the close agreement between experimental and literature values confirms the preservation of the DMT molecular structure, suggests minimal contamination, and demonstrates the effective isolation of the compound, since the presence of other molecules would significantly alter the intensity and position of the absorption bands.

### 3.7 Thermogravimetric Analysis (TGA)

Figure 6 presents the TGA and DTG curves for the DMT crystals obtained from sample 2C. Thermogravimetric analysis (TGA) of the crystals extracted from the stem bark via Methodology 2 revealed a single thermal degradation event, starting at 139.93 °C and ending at 231.42 °C, accompanied by a significant mass loss of 97.2%. The absence of prior thermal events, especially in the temperature range near DMT′s melting point (∼40 °C), indicates that no residual solvents remain trapped within the crystalline matrix, thereby supporting the successful isolation of the compound. These thermal characteristics align with previous findings by Amariz et al.,^51^ who reported initial mass loss attributed to the release of residual solvents, suggesting that physical instability is potentially associated with the presence of impurities. Therefore, the TGA results in this study demonstrate that the DMT crystals possess minimal or negligible contaminant content, a critical factor for biomedical and pharmaceutical application.

**Figure 6:**
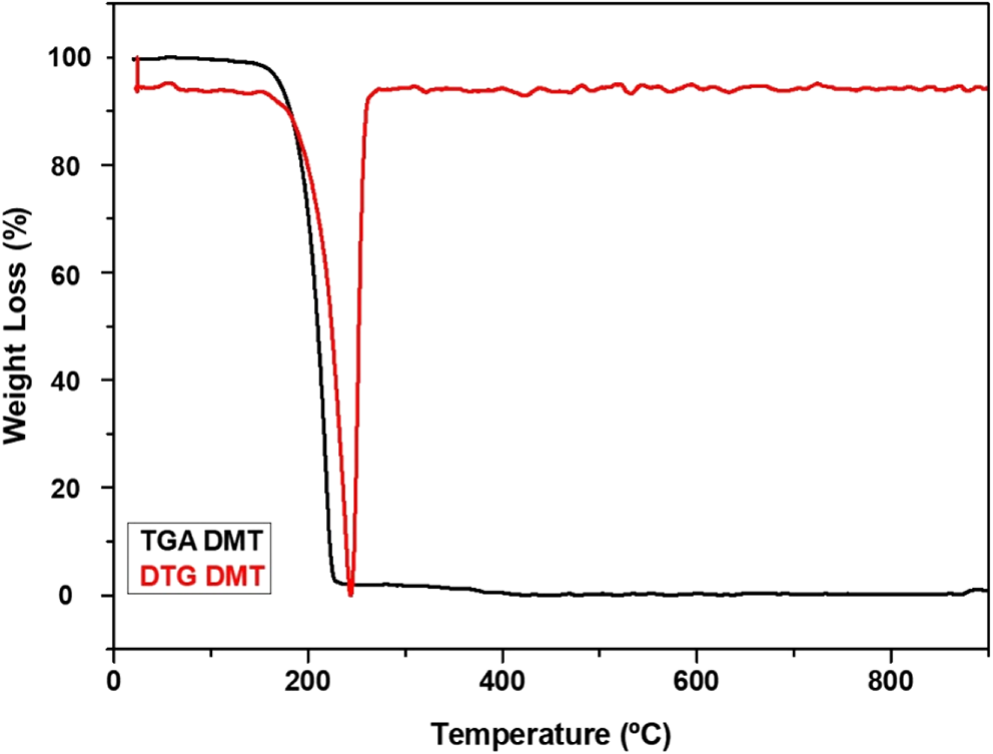
TGA/DTG curves of DMT at a heating rate of 10 °C/min from 25 to 800 °C under a nitrogen flow (50 mL/min).

### 3.8 High-Performance Liquid Chromatography with Diode Array Detection (HPLC-DAD)

High-performance liquid chromatography with diode array detection (HPLC-DAD) was used to qualitatively confirm the presence of DMT. As shown in Figure 7, the analysis demonstrated high selectivity and specificity, evidenced by a single, well-resolved peak with sharp intensity and clear separation at a retention time of 11.81 min.

**Figure 7:**
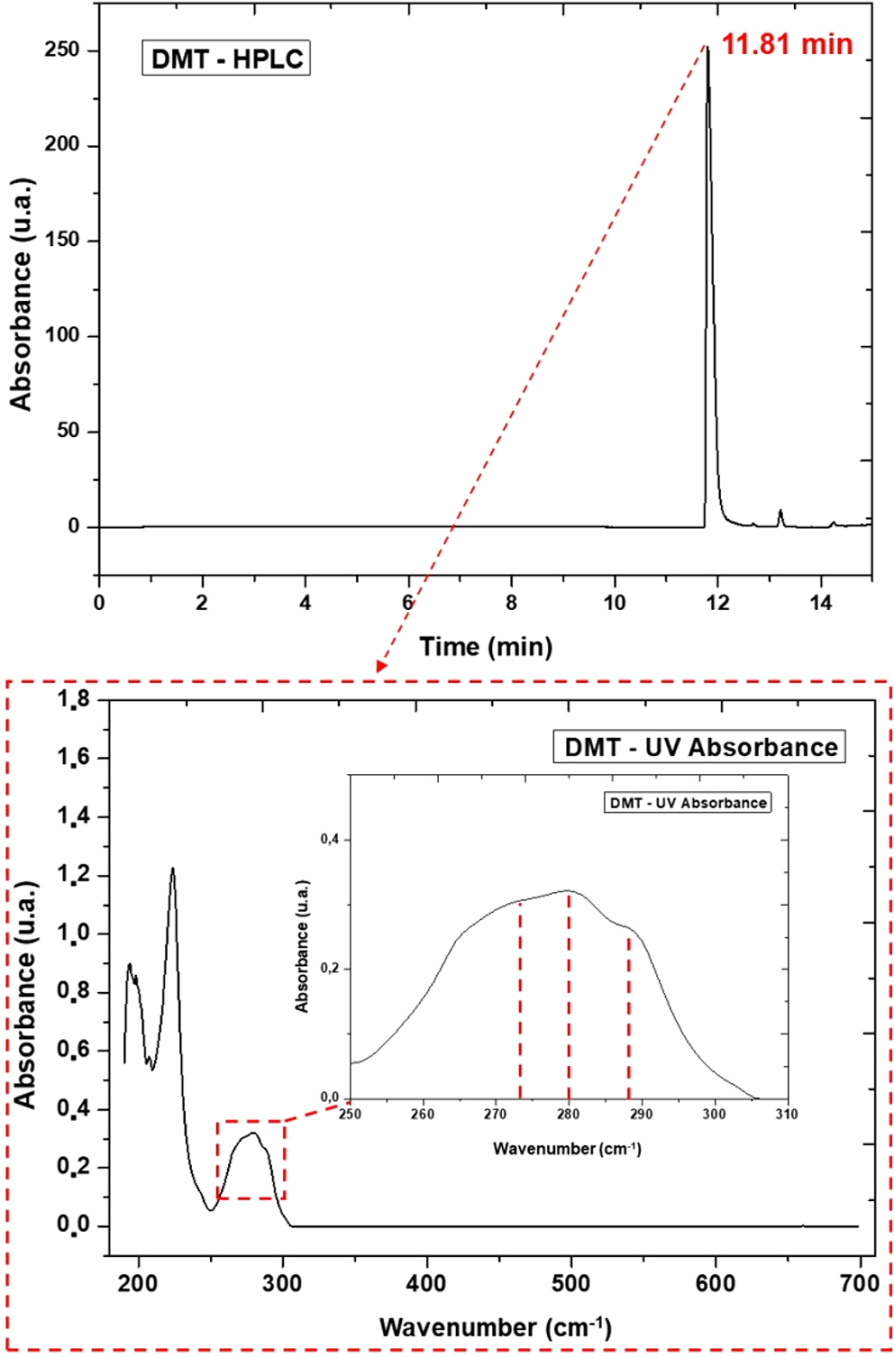
Chromatogram of isolated DMT crystals obtained by HPLC-DAD (above) and absorption spectra for DMT (below), highlighting the absorption region between 270 and 300 nm.

This result is particularly noteworthy, as the observed retention time aligns closely with that reported by Amariz et al.,^51^ who detected DMT at approximately 9 min under comparable chromatographic conditions. Minor variations in operational parameters, such as the mobile phase gradient rate, account for the slight differences in retention time. Nevertheless, the consistent identification of DMT within a compatible retention window underscores the robustness and reliability of HLPC-DAD for characterization of tryptamine alkaloids.

Furthermore, the DAD detector recorded absorption bands at 275, 280, and 288 nm, over the corresponding retention time, closely matching the data reported by Gaujac,^26^ who documented a maximum absorbance of DMT at 275 nm and similar profiles for tryptamine at 290 nm. This UV absorption bands are characteristic of the indole nucleus present in DMT, confirming the presence of the substituted indole chromophore. The observed signals correspond to typical π→π transitions of the indole moiety, which strongly absorb in the UV region. The clean, single-peak profile combined with this spectral signature further supports the high purity of the isolated compound.

Overall, the HPLC retention time and corresponding UV spectral features provide converging evidence for the structural identity of DMT isolated from the bark. The characteristic indole absorption confirms that the isolated substance retains its chromophoric integrity, while the absence of secondary peaks or unexpected signals underscores the effectiveness of the extraction and purification protocol in yielding high-purity material.

The presence of a single, well-defined peak with its characteristic spectral profile, and the absence of secondary signals, reinforces both the purity of the isolated DMT and the efficiency of the extraction protocol. This level of purity is essential for applications demanding stringent quality, including pharmaceutical use, toxicological assessments, and pharmacokinetic studies.

### 3.9 Gas Chromatography–Mass Spectrometry (GC–MS)

The GC-MS analysis, presented in Figure 8, shows a single distinct peak at a retention time of 16.4 min, which was attributed to DMT. Identification was confirmed through comparison with the NIST database, showing a 88% matching with the reference spectrum. While slightly lower than the 98% similarity reported by Gaujac^26^ using the Wiley library, with retention time of 21.2 min, the discrepancy can be attributed to variations in methodological parameters, including the column type, temperature program, and split injection mode (1:30). These factors influence compound volatilization and chromatographic separation, resulting in different retention times without compromising the specificity of the identification. Furthermore, the NIST library search revealed 20 high-similarity hits, all identified as DMT, confirming the unequivocal identification of the analyte.

**Figure 8:**
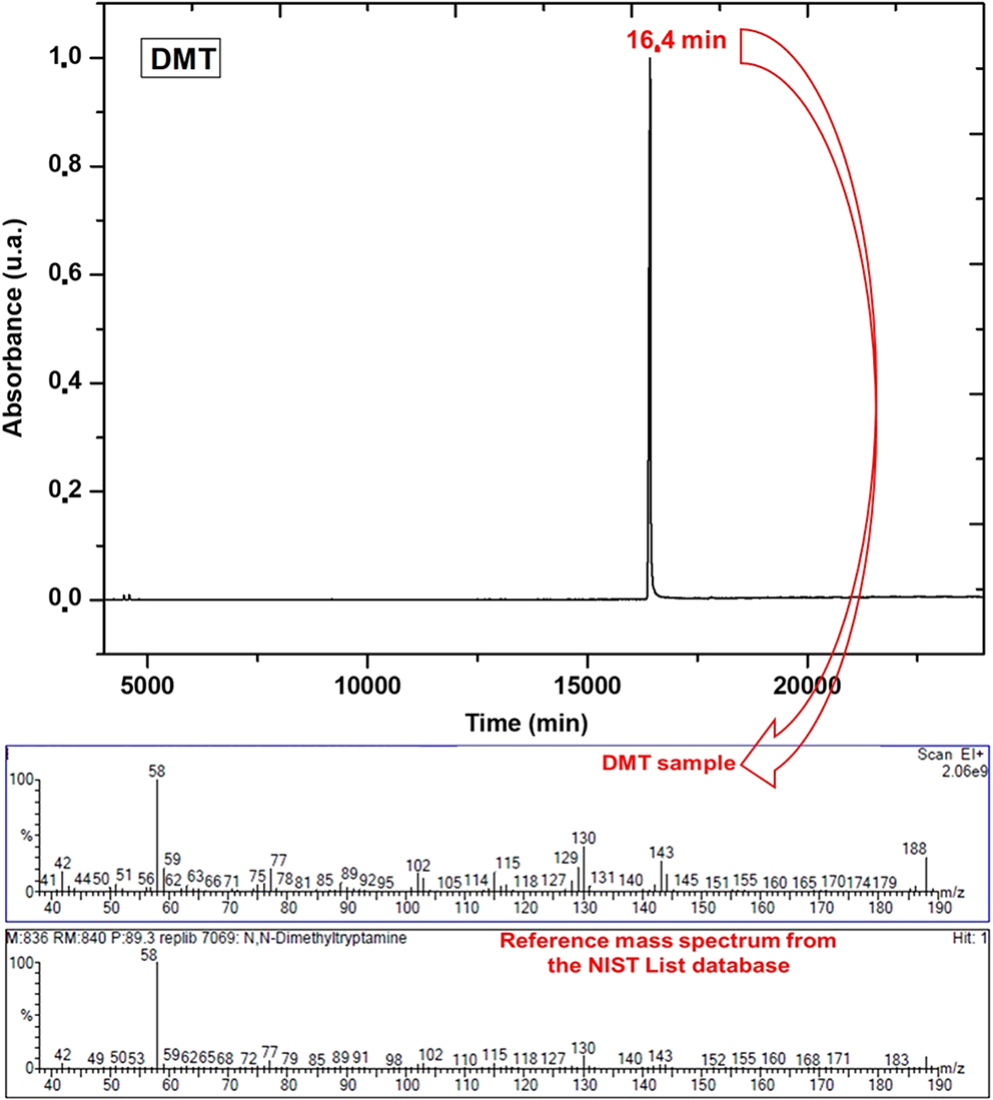
Gas chromatography chromatogram showing the DMT peak at 16.4 min. Upper panel displays the observed fragmentation spectrum of the compound. Lower panel presents the reference fragmentation spectrum from the NIST library, enabling direct comparison and confirming the identity of DMT.

The fragmentation spectrum exhibited the characteristic ions of DMT, including the base peak at *m*/*z* 58, along with ions at *m*/*z* 130 and 188, consistent with literature reports by Gaujac^26^ and entries in the NIST database. The *m*/*z* 130 ion, linked to fragmentation of the substituted indole ring, is particularly relevant structural confirmation of DMT, also highlighted by Gaujac. The peak at *m*/*z* 188 corresponds to the molecular ion, supporting the molecular integrity and the efficiency of electron impact ionization (70 eV). The high spectral resolution, combined with the absence of extraneous peaks in the chromatograms, indicates both high sample purity and the suitability of the analytical protocol used for detecting tryptamine alkaloids, which is a critical criterion for reliability in pharmacological and toxicological applications.

### 3.10 Cytotoxicity

The cytotoxicity assay results, shown in Figure 9, indicate that DMT crystals isolated from *M. tenuiflora* exhibit a cell viability profile largely consistent with the biocompatibility criteria established by ISO 10993–5:2009. Figure 9 displays both the raw optical density readings for each experimental replicate and the calculated average cell viability, providing a comprehensive view of the data distribution. Exposure to DMT at concentrations of 5, 25, 50, and 75 μg/mL resulted in cell viabilities of 92%, 88%, 71%, and 71%, respectively. At the highest tested concentration (100 μg/mL), a more pronounced reduction in viability was observed, with values reaching 53 ± 21%. The L929 fibroblast cells were maintained under standard culture conditions for 24 h prior to exposure, and cytotoxicity was assessed after 24 h of contact with the test DMT solutions. Although no previous studies reporting DMT cytotoxicity under ISO 10993–5:2009 were identified, the obtained results demonstrate compliance with the standard for concentrations up to 75 μg/mL (≥70% viability), indicating acceptable cell viability within this range.

**Figure 9:**
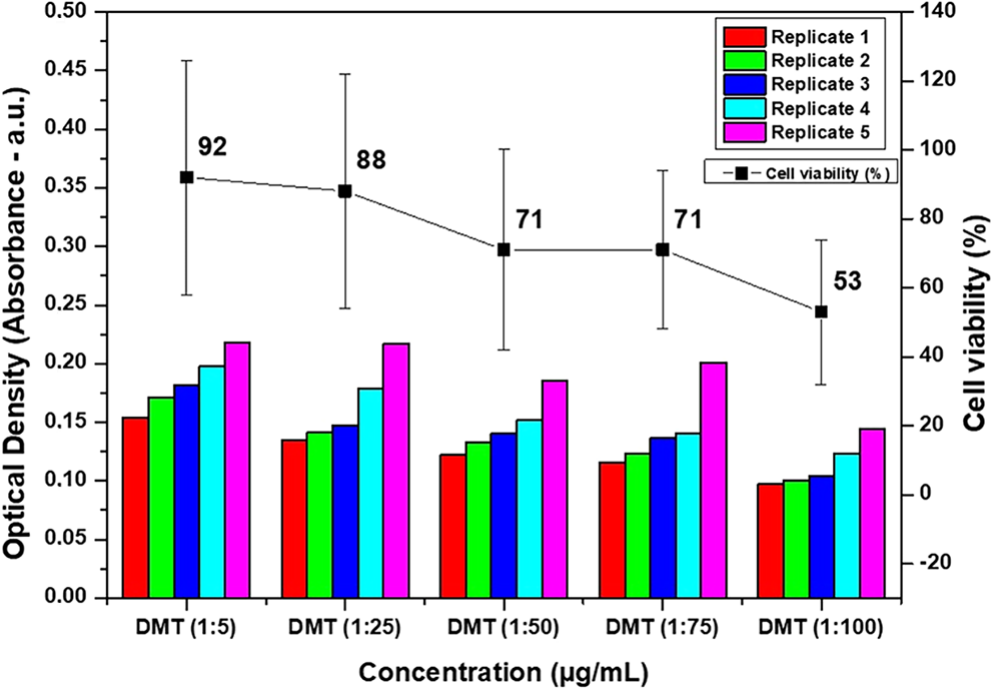
Cytotoxicity of DMT isolated from *M. tenuiflora*.

Although this last value falls below the ISO 10993–5 acceptability threshold, this result should be contextualized and interpreted in light of the occurrence of unusual behavior observed during sample preparation. Notably, the rapid and complete dissolution of DMT crystals in phosphate-buffered saline (PBS), irrespective of concentration, producing visually turbid solutions. This physicochemical behavior likely contributed to the higher variability among replicates, particularly notable at 100 μg/mL as reflected in the bar graph. While this phenomenon has not been reported in other consulted studies, it may indicate a physicochemical peculiarity of the isolated DMT, possibly related to its high affinity for the aqueous media and rapid solubilization. This behavior could have increased local bioavailability, leading to enhanced effective cellular exposure, and contributing to the more pronounced reduction in cell viability at the highest tested concentration.

This characteristic suggests an elevated bioavailable fraction of the substance, potentially increasing the effective cellular exposure and thereby amplifying the cytotoxic effects observed at higher concentrations. Consequently, it is plausible that the measured cytotoxicity profile partly reflects experimental overexposure, which may differ from the response under controlled physiological conditions.

Despite this methodological limitation, at concentrations close to those typically proposed for therapeutic delivery systems, the isolated DMT clearly exhibited minimal residual cytotoxicity, maintaining cell viabilities above 85%. Although specific concentration ranges for sublingual DMT formulations are lacking, pharmacokinetic studies of other administration routes indicate that psychopharmacological effects are typically observed with doses of 20–60 mg via vaporization, 0.2–1 mg/kg via intramuscular injection, and approximately 1.3 mg/min via continuous intravenous infusion, yielding maximum plasma concentrations around 15.7 ng/mL, as reported by Barker.^55^ These data suggest that effective sublingual formulations would likely involve concentrations well below those associated with *in vitro* cytotoxicity, reinforcing the safety of the compound under the tested conditions.

This performance indicates a biologically relevant safety margin, positioning the obtained compound as a promising candidate for medical applications targeting psychiatric disorders, particularly within the emerging field of psychedelic-assisted psychotherapy. The rapid solubilization of DMT, initially perceived as a potential experimental drawback, can be leveraged pharmaceutically as a strategic advantage for designing delivery systems with controllable and predictable kinetic profiles. Thus, despite the noted experimental limitations, the data support the viability of using DMT as a basis for the development of innovative, safe medical interventions capable of addressing the increasing therapeutic demands in mental health.

## 4 Conclusions

This comparative study successfully identified an optimized protocol for the extraction of DMT from *M. tenuiflora*, with sample 2C (stem bark, Methodology 2) showing the most favorable performance. This superiority is attributable not only to its higher extraction yield (3.45%), a significant improvement over the 2.11% reported in prior optimization studies such as Amariz et al.,^15^ but also to its rich phytochemical profile, notably diverse in alkaloids, tannins, and flavonoids. The validation of the high purity and structural integrity of the isolated DMT was performed using a robust analytical platform. HPLC-DAD and GC-MS analyses provided structural and quantitative validation, demonstrating high specificity and 88% spectral similarity with the NIST library, expanding the foundational GC-MS methodology established by Gaujac.^22^ Physicochemical characterization further corroborated the material’s quality: SEM analysis revealed the transition from an initial amorphous state to well-defined prismatic crystals upon recrystallization; EDS confirmed elemental purity, with a 76.03% carbon and 23.97% nitrogen; FTIR validated presence of characteristic of indolic alkaloids functional groups; and TGA demonstrated thermal stability up to 135 °C, a crucial parameter for pharmaceutical processing. Regarding the biomedical potential, cytotoxicity assays indicated that cell viability remained above 85% at therapeutically relevant concentrations, supporting the biocompatibility and safety of the isolated compound for potential biomedical applications.

Overall, this work establishes a benchmark for obtaining high-purity, pharmacologically viable DMT from *M. tenuiflora*. By integrating an efficient extraction method with thorough analytical characterization and preliminary safety evaluation, the study provides a validated framework for developing standardized formulations suitable for future clinical research.
